# Effects of cheerleading practice on advanced glycation end products, areal bone mineral density, and physical fitness in female adolescents

**DOI:** 10.3389/fphys.2022.954672

**Published:** 2022-09-05

**Authors:** Lijun Wang, Hongli Zhang, Tuo Xu, Jing Zhang, Yuanyuan Liu, Yue Qu

**Affiliations:** Physical Education Institute of Shaanxi Normal University, Xi’an, China

**Keywords:** cheerleading practice, female adolescents, areal bone mineral density, advanced glycosylation end products, physical fitness

## Abstract

**Background:** Exercise has been widely reported to promote bone health, but it is unknown whether is associated with a reduction in advanced glycosylation end products (AGEs). This study aimed to investigate the effects of 14 weeks of cheerleading exercise on areal bone mineral density (aBMD) and AGEs.

**Methods:** In this study, 46 female teenagers (age, 19.52 ± 1.21 years; body mass index, 20.15 ± 2.47 kg/m^2^) were randomly divided into a cheerleading group (CHE, *n* = 21) and a control group (CON, *n* = 25). The CHE group was subjected to cheerleading practice twice a week for 14 weeks; the CON group maintained their daily routine. Dual-energy X-ray absorptiometry was used to measure aBMD, and autofluorescence (AF) values were used to reflect AGEs. Physical fitness testing all-in-one machines are used to test body composition, cardiorespiratory fitness, muscle fitness and flexibility. A mixed ANOVA model was used to examine the effect of the intervention on each outcome. A multiple mediation model with covariates for physical activity and eating behaviors was performed to explore the mediators between cheerleading exercise and aBMD.

**Results:** After 14 weeks of cheerleading practice, 1) aBMD increased significantly in both groups with significantly higher increases in the CHE group (*p* < 0.05). 2) AGEs significantly decreased in the CHE group (−2.7%), but not in the CON group (*p* > 0.05). 3) Vertical jumps and sit-ups significantly increased in the CHE group (*p* < 0.05), but not in the CON group (*p* > 0.05). 4) ΔAF values was significantly negatively correlated with *Δ* aBMD (*r* = −0.302, *p* < 0.05). 5) ΔAF values mediated the effect of exercise on the aBMD (indirect effect: 0.0032, 95% CI 0.0002–0.0079).

**Conclusion:** Cheerleading practice improved aBMD and physical fitness and reduced AGEs accumulation in female adolescents. The effect of exercise on aBMD was partially mediated by AGEs.

## Introduction

Adolescent bone health is an important public health issue. In recent years, adolescent bone health has been challenged by a range of lifestyle changes, such as reduced physical activity, obesity, High-fat and sucrose diet, and eating disorders (Hallal et al., 2006; Lorincz et al., 2010; Hills et al., 2011). Low bone mineral density is due to a failure to increase peak bone mass in adolescence and a decrease in bone mass in young adulthood. Studies have shown that sedentary behavior, obesity, and eating disorders can contribute to low bone mineral density in adolescents and are important risk factors for adolescent bone health (Pelegrini et al., 2020; Zuckerman-Levin et al., 2014; Pollock, 2015). Female adolescents may be at greater risk for bone health as most adolescent girls do not meet physical activity guidelines and are more likely to have an eating disorder (Treuth et al., 2007; Bland et al., 2020; Thornton and Gordon, 2017). Therefore, it is important to take care of adolescent bone health issues, especially for female adolescents.

The harmful effects of Advanced glycosylation end products (AGEs) on bone health have been one of the greatest concerns of researchers in recent years. (Sanguineti et al., 2014; Lamb et al., 2018). AGEs are a complex and heterogeneous group of compounds (Semba et al., 2010; Vistoli et al., 2013). AGEs have a significant impact on human health (Yamamoto and Sugimoto, 2016). Researchers investigated 9,203 patients, whose bone mineral density (BMD) was assessed by quantitative ultrasound techniques, and their AGEs were assessed by measuring skin autofluorescence (AF) values, suggesting that the accumulation of AGEs was a detrimental factor for bone health (Tabara et al., 2019). AGEs could affect bones throughout a person’s entire life span (Vashishth et al., 2001; Thomas et al., 2018). Studies have found that the accumulation of AGEs might be a risk factor for osteoporosis and fractures in the older population (Asadipooya and Uy, 2019). Baxter et al. (Baxter-Jones et al., 2011) showed that childhood and adolescence are prime periods for bone mineral accumulation, with stable peak bone mass levels being reached during the first 2 decades. The pentosidine, a type of AGEs, was negatively correlated with the bone mineral content in adolescents suggesting that the accumulation of AGEs may affect peak bone mass in young people (Kindler et al., 2019). AGEs might affect bone via the following mechanisms. AGEs physically affect the properties of bone material and bone mass through their accumulation in collagen fibers (Yamamoto and Sugimoto, 2016). In addition, AGEs adversely affect human interstitial bone marrow stem cells and play a harmful role in the pathogenesis of skeletal disorders (Kume et al., 2005).

Exercise can be a non-medical intervention to promote bone health. The frequency and intensity of exercise is important in influencing the bone health of adolescents (Ishikawa et al., 2013; Rowlands et al., 2020). Appropriately designed aerobic exercise and resistance training can improve bone health in young people (Greene et al., 2006; Lloyd et al., 2014). Cheerleading is an aerobic dance sport that includes many aerobic and self-weight training elements and has been found to benefit the development of cardiorespiratory endurance and strength in adolescents (Krivoruchko, 2018). Studies have shown that aerobic dance are beneficial for improving women’s bone health (Na and Kritpet, 2015; Yang et al., 2022). The beneficial effects of exercise on bone may be related to appropriate mechanical stress stimulation (Li et al., 2019) and the secretion of muscle-derived cytokines (Colaianni et al., 2019).

Exercise may also be an effective way to reduce AGEs. Karine et al. (Rodrigues et al., 2018) found that exercise reduced AGEs levels in inactive patients with acquired immunodeficiency virus (HIV), suggesting that short-term moderate-intensity aerobic training could reduce AGEs levels in the body. Reduced accumulation of AGEs in the body by exercise may be associated with decreased hyperglycemia (Reddy et al., 2019) and increased clear efficiency (Shen et al., 2020). However, it is unclear whether cheerleading, one of the more popular sports among female adolescents, can reduce the accumulation of AGEs and promote bone health in female adolescents. It is also unclear whether the improvement in bone health from exercise is associated with a reduction in the accumulation of AGEs in the body. Few studies explore the effects of exercise on bones and AGEs simultaneously.

In general, most of the research on the damage to bone health caused by AGE accumulation has been epidemiological (Li et al., 2012; Rodrigues et al., 2018; Tabara et al., 2019) and animal studies (van Dongen et al., 2021), but there has been a lack of research on the effects of exercise on AGE accumulation and bone health in humans. Therefore, this study explores the effects of cheerleading on AGEs, aBMD, and physical fitness in female adolescents, and provides a practical basis for intervention strategies to improve the overall health of female adolescents.

## Materials and methods

### Participants

**FIGURE 1:**
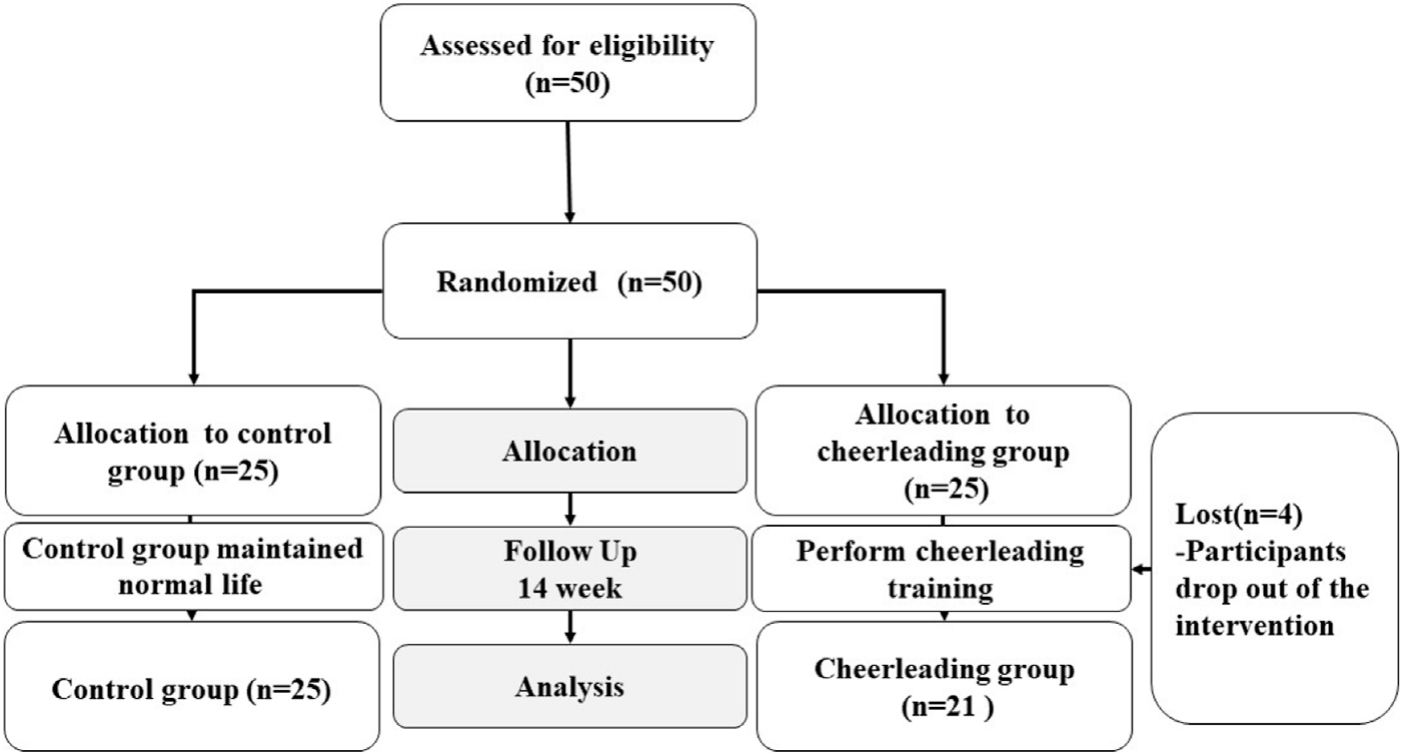
Participants’ recruitment and intervention flow diagram.

From March 2021 to July 2021, A total of 50 female adolescents were recruited as participants, and they were randomly divided into a cheerleading group and a control group (CON, *n* = 25). Four participants dropped out of cheerleading practice, three because of inability to persist and one because of relocation (CHE, *n* = 21) (Figure 1). All participants had never been trained in cheerleading before. Participants were informed of the whole process of the experiment and the experimental procedure is in accordance with the Declaration of Helsinki. The study was approved by the Ethics Committee with approval number 202216003.

### Study design and procedures

The CHE group conducted the cheerleading practice for 14 weeks in two different ways. A: the intervention took place every Monday in the gym; B: every Saturday they trained independently at home via the internet, practicing the cheerleading motions they had learned in class. The training recordings were transmitted through photos and videos to chat software, which was monitored and checked by the researcher. The duration of each training session was 100 min. The control group lived in the same environment as the CHE group, maintained their daily routine and received a health education before the start of the experiment. The volume, intensity and density of a typical training session have been shown in Table 1. All practice schedules can be found in the supplementary material. Height, weight, body mass index (BMI), body composition, aBMD, and AGEs levels were measured before and after the intervention. All tests were carried out by trained professional researchers.

**TABLE 1:** A typical cheerleading practice schedule.

Intervention Content	Density	Intensity	Equipment	Time (min)
Preparation exercise	Preparation exercises 1 set, 2 reps a movement	50–60% HR_max_	Playground	5
1) Dynamic preparation activities				
(neck, shoulder, spine, pelvis, rump, knee, ankle)				
2) Arm control exercises: Lateral arm raises, small up and down arm vibrations, small back and forth arm vibrations, lateral arm raises in clockwise circles, lateral arm raises in counterclockwise circles, etc. kicking exercises: front kick, side kick, beside kick, Sucking leg jump, back stomp run, trot pony jump.	Arm control and kicking exercises 2 sets of 4 reps of one movement	55–65% HR_max_	flower ball	15
3) Learn the 8 hand positions: (upper M, lower M, W-movement, high V, inverted V, T-movement, diagonal movement, short T-movement) learn the basic leg steps:(stand with legs together, stand with legs apart, lunge standing)	Divide the new movements into stages, gradually learning 8 new hand positions and basic leg steps, practicing 4 sets of 8 reps of each movement	55–65% HR_max_	flower ball	40
4) Practice the basic hand positions and leg movements learned in Part 3	Dance the basic hand positions and leg movements learned in part 3 for 4 consecutive 8 beats	65–75% HR_max_	flower ball	30
5) Relaxation exercises: stretching the large arms, small arms and wrists. Stretching the front and back of the legs and the outside of the legs	Relaxation exercises in 2 sets of 4 reps for each movement	50–60% HR_max_	Playground	10

### Physical activity, eating behavior, exercise enjoyment, mental health measurement

A short version of the International Physical Activity Questionnaire (IPAQ) (Lee et al., 2011) was used to assess the daily energy expenditure of participants. This self-administered questionnaire consists of 7 items. Six of the items were about the time spent on vigorous exercise, moderate exercise, and walking. The last question was about the amount of time spent sitting each day.

Eating behavior was measured using the Three-Factor Eating Questionnaire (TFEQ) (de Lauzon et al., 2004), which measures participants’ eating habits in three dimensions: cognitive restriction, uncontrolled eating, and emotional eating. The Eating Behavior Scale which consists of 21 items is a 4-point response scale (absolutely correct/basically correct/basically incorrect/absolutely incorrect). The total score was obtained by summing the three dimensions’ scores.

Sports enjoyment was measured using the Physical Activity Enjoyment Scale (PACES) (Gnambs and Staufenbiel, 2018; Teques et al., 2020), which consists of 16 items.

### Anthropometric measurements

Researchers first used the Physical Fitness Assessment System TA106 (IDONG; Shenzhen Taishan Sports Technology Co., Ltd.,). to measure height and weight. During the test, the participant stood naturally with their hands down at their sides and their eyes looking straight ahead, and the results were automatically saved and recorded. Body composition was measured using a bioelectrical impedance method (Inbody 230, Biospace Inc., Seoul, Korea). During the test, participants were asked to stand naturally, keeping the spine in a neutral position. Participants held electrode rods in their hands and took off their shoes and socks, placing their bare feet on metal electrodes to ensure direct contact between the skin and the metal electrodes. The percentage of body muscle and fat will be automatically saved and recorded.

### Bone parameters measurement

Areal bone mineral density at the distal end of the radius was measured using dual-energy x-ray absorptiometry Bone mineral Densitometer DXA-IMAX (Kang Rong Xin Intelligent Medical System Co., Ltd., Xi’an, China), which is examined and calibrated before each test to ensure accuracy. Calibration was performed using a phantom with the same shape as the real bone until the measured aBMD reading was the same as the actual labeled value of the phantom, then the calibration was successful. The coefficient of variation of aBMD measured by repeated measurements on the same day and multiple days using a 4 mm phantom was ≤0.01. The dominant hand of all participants is the right hand. The participant sits in a specific position and uniformly places her right hand into the aBMD testing device, placing the end of the wrist at the infra-red crossing marked on the device as required, with the eyes looking straight ahead. The test result indicators aBMD, Bone mineral content (BMC) and will be automatically saved and recorded.

### AGEs measurement

Autofluorescence (AF) value, an indicator of the accumulation of AGEs in the body, was measured by the Advanced Glycation End Product Reader for AGEs READER (Diagnoptics Technologies B.V Co., Ltd., Netherlands). This method is so simple as to be widely used (Meerwaldt et al., 2004). Participants were asked to not use skin care products such as sunscreen on the arm area before measurement. Calibration is carried out before each test using the manufacturer’s matching kit until the calibration is successful with an autofluorescence value of 2.65. Participants place the forearm of the right hand on the device and the average of three consecutive measurements is taken to determine the AF level. The test was conducted with the participant seated in a designated position, with the palm side of the lower arm approximately 10 cm in front of the elbow joint pressed against the excitation point of the device, and the AGEs READER excitation light source illuminating approximately 1 cm 2 of skin on the palm side of the lower arm to measure AF at a peak wavelength of 370 nm. The measurement should be taken on normal skin, avoiding visible blood vessels, scars, lesions, or other skin abnormalities. The measured data is automatically stored and recorded by the skin fluorescence detector.

### Physical fitness measurements

Physical fitness (PF) tests were performed using Health Fitness Instrument TA106 (IDONG; Shenzhen Taishan Sports Technology Co., Ltd.). The main tests include vertical jump, sit-ups, balance, sit and reach test, systolic and diastolic blood pressure. Throughout the test, participants followed the instructions of the staff and completed each step correctly. In the vertical jump test, participants were asked to jump vertically upwards with their hands naturally down and knees slightly bent, jumping twice to take their highest value. In the sit-up test, participants were asked to start in a flat position, with their hands by their ears, and finish once with their elbows touching their knees. In the balance test, participants close their eyes at the instruction of the machine, naturally lift either foot and naturally lower their arms until they cannot maintain their balance, the machine automatically records the time taken to maintain standing and the maximum result is recorded after two tests. In sit and reach tests, participants were asked to sit with knees straight, upper limbs extended and arms straight, with the participant sitting on the machine with the entire upper body bent forward so that the fingers reached the furthest scale on the device scale to obtain the best result. The electronic spirometer was used to measure the vital capacity of the participants. The vital capacity index was obtained using vital capacity divided by body weight. The step test was used to measure the participants’ cardiorespiratory fitness. Participants followed the beats of the metronome and went up and down the steps at a rate of 30 steps/min for a total of 3 min and then rested for 4 min. Heart rate during rest was recorded using photoplethography and the step index was calculated using the following formula.

Step index=180×100/2(P1+P2+P3)

P1, P2 and P3 are the heart rates for the three recovery periods of 1–1 min 30 s, 2–2 min 30 s and 3 min–3 min 30 s after exercise.

### Statistical analysis

The sample size required for this study was calculated by G Power version 3.1.9.7 (Kiel University, Germany). It was calculated that a sample size of at least 44 participants was required to achieve 95% statistical power and this study met the minimum sample requirement. All data were tested for normal distribution by Kolmogorov-Smirnov statistic and descriptive statistics were used as mean ± standard deviation. Independent samples *t*-test was used to test for differences in baseline indicators between the two groups. A mixed ANOVA model (within-subjects factor: 2 time × between-subjects factor: 2 groups) was used to explore the effects of exercise on AGEs, bone, and PF, and post hoc analysis was performed with Bonferroni. The effect sizes were expressed using *η*_p_^2^. Spearman correlation analysis was used to examine the association between the aBMD and the change in AF values.

A parallel multi-mediator model was performed using the PROCESS macro in SPSS to examine whether the effect of exercise on aBMD was directly and/or indirectly carried through changes in body composition (muscle mass and body fat percentage), AF values, and physical fitness. When the independent variable (exercise) entered the model, it was set as a dummy variable (no exercise = 0; exercise = 1). The mediating variables were selected as those with significant differences between groups after exercise. Physical activity and eating behaviour at baseline were added to the mediation model as covariates. Since previous studies have shown that these two covariates are closely associated with the status of aBMD and AF values (Waqas et al., 2020; Del et al., 2021). The bootstrap confidence interval (CI) method was used to infer direct, indirect, and overall effects in the mediated model, with confidence intervals not containing 0 considered significant, and the bootstrap sample was 5,000.

A *p*-value < 0.05 was considered statistically significant. SPSS version 23.0 (Provided by SPSS Inc., Chicago, Illinois, United States) software was used for statistical analysis.

## Results

### Participants

A total of 46 female adolescents were eventually included in this study. Participants had a mean age of 19.52 ± 1.21 years, a mean height of 162.73 ± 5.71 cm, and a mean BMI of 20.15 ± 2.47 kg/m^2^. Table 2 shows the baseline characteristics of the participants, with no significant differences between the two groups in height, weight, BMI, physical activity, eating behavior, exercise enjoyment (*p* > 0.05). Table 3 shows the pre-intervention data for aBMD, BMC, AGEs, and PF, none of which were significantly different between the two groups before the intervention (*p* > 0.05).

**TABLE 2:** Basic participant characteristics (mean ± SD).

	CHE Group	CON Group	Total
Age (year)	18.48 ± 0.75	20.40 ± 0.71	19.52 ± 1.21
Height (cm)	164.01 ± 5.22	161.67 ± 5.97	162.73 ± 5.71
Weight (kg)	55.21 ± 7.79	52.00 ± 8.07	53.47 ± 8.02
BMI (kg/m^2^)	20.51 ± 2.68	19.84 ± 2.30	20.15 ± 2.47
Systolic pressure (mmHg)	112.80 ± 10.80	111.20 ± 9.35	111.93 ± 9.95
Diastolic pressure (mmHg)	71.50 ± 8.45	73.28 ± 7.44	72.47 ± 7.78
TFEQ-21	49.26 ± 6.67	50.86 ± 8.55	50.07 ± 7.65
UE	22.81 ± 3.80	23.57 ± 4.73	23.20 ± 4.28
CR	13.70 ± 3.56	23.57 ± 4.73	23.20 ± 4.28
EE	12.74 ± 9.65	13.07 ± 1.98	12.91 ± 1.82
PACES	37.96 ± 9.65	35.50 ± 10.01	36.71 ± 9.82
IPAQ (met/min·week)	2,204.27 ± 1703.06	1985.84 ± 1364.10	2093.07 ± 1529.39
Low PA level	1 (4.8%)	1 (4.0%)	2 (100.0%)
Moderate PA level	16 (76.2%)	20 (80.0%)	36 (100.0%)
Vigorous PA level	4 (19.0%)	4 (16.0%)	8 (100.0%)

Abbreviations: CHE, cheerleading group; CON, control group; BMI, body mass index; TFEQ: three-factor eating questionnaire; UE: uncontrolled eating; CR: cognitive restraint; EE: emotional eating; PACES: physical activity enjoyment scale; IPAQ: international physical activity questionnaire; PA: physical activity.

**TABLE 3:** AF, aBMD, and physical fitness in the pre-and post-intervention.

	CHE Group				CON Group				Interaction	
	Pre	Post	*P*	ES	Pre	Post	*P*	ES	*P*	η^2^_p_
aBMD (g/cm^2^)	0.37 ± 0.02	0.38 ± 0.01	0.000	0.396	0.37 ± 0.01	0.37 ± 0.01^a^	0.001	0.228	0.134	0.050
BMC (g)	4.57 ± 0.62	4.68 ± 0.62	0.123	0.053	4.48 ± 0.44	4.51 ± 0.50	0.552	0.008	0.454	0.013
AF	1.77 ± 0.06	1.72 ± 0.06	0.004	0.175	1.74 ± 0.08	1.77 ± 0.09^a^	0.096	0.062	0.001	0.209
Muscle mass (kg)	37.76 ± 2.89	38.57 ± 2.58	0.002	0.211	36.89 ± 3.54	36.71 ± 3.45	0.356	0.020	0.003	0.187
%BF	25.60 ± 6.23	23.99 ± 6.13	0.000	0.291	23.36 ± 6.23	22.05 ± 6.13	0.001	0.249	0.584	0.007
VJ (cm)	23.73 ± 4.66	26.04 ± 4.12	0.004	0.178	21.94 ± 3.67	22.50 ± 4.30^a^	0.417	0.015	0.084	0.066
SRT (cm)	15.83 ± 6.22	14.74 ± 6.18	0.173	0.043	13.66 ± 5.52	12.98 ± 5.21	0.334	0.022	0.705	0.003
Sit-ups (reps/min)	32.40 ± 7.41	34.70 ± 7.82	0.062	0.077	28.28 ± 7.42	28.48 ± 6.90^a^	0.856	0.001	0.203	0.037
Balance (s)	35.80 ± 34.71	53.47 ± 37.79	0.019	0.118	36.64 ± 36.26	53.23 ± 37.10	0.017	0.123	0.914	0.000
VCI (ml/kg)	55.98 ± 10.12	57.04 ± 8.57	0.456	0.013	56.08 ± 8.73	57.07 ± 9.50	0.446	0.013	0.942	0.000
Step test index	55.21 ± 13.01	49.14 ± 10.42	0.000	0.281	60.09 ± 11.36	52.96 ± 9.04	0.000	0.391	0.596	0.006

Abbreviations: CHE, cheerleading group; CON, control group; aBMD, areal bone mineral density; BMC, bone mineral content; AF, autofluorescence; %BF, body fat percentage; VJ, vertical jump; SRT, sit and reach test; VCI, vital capacity index; Interaction: interactive effects; ES, effect size.

a Indicates a significant difference compared to the post-exercise CHE, group.

Autofluorescence (AF) value, an indicator of the accumulation of advanced glycosylation end products in the body.

### Bone parameters

**FIGURE 2:**
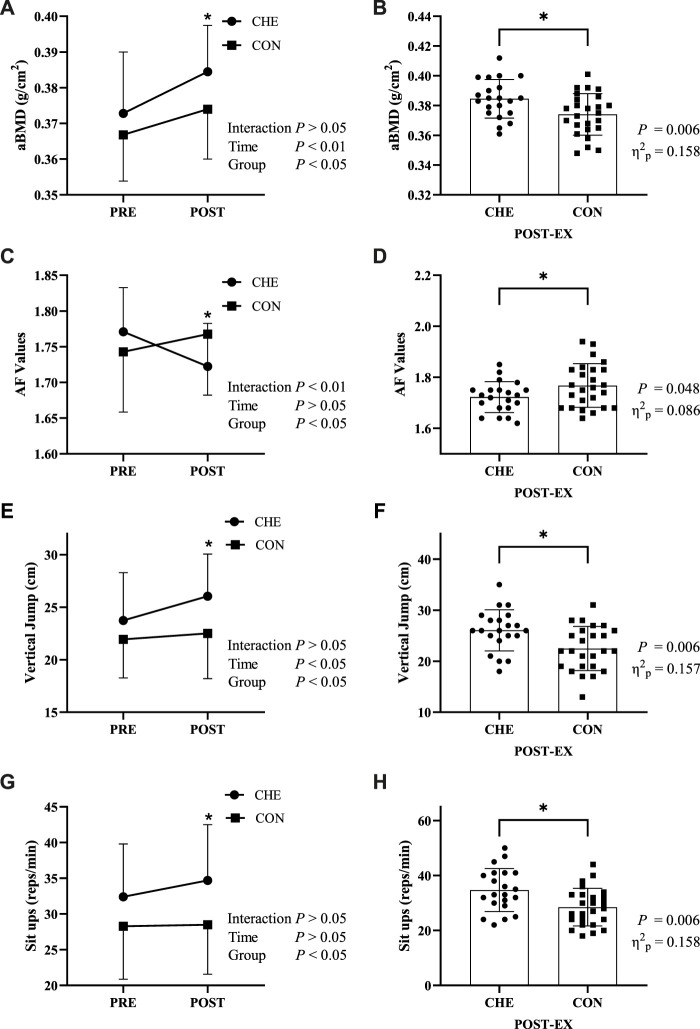
Changes in indicators before (PRE) and after (POST) the intervention in the cheerleading group (CHE) and the control group (CON). **(A,B)** areal bone mineral density (aBMD); **(C,D)** auto-fluorescence (AF) Values; **(E,F)** vertical jump; **(G,H)** sit-ups. * indicates a significant difference between the CHE and CON groups. Interaction: interactive effects; Time: time main effect; Group: group main effect.

For aBMD there was no significant time × group interaction effect (*p* = 0.134, η_p_^2^ = 0.050) (Figure 2A). Simple effect for time showed that aBMD increased significantly in both the CHE group (*p* = 0.000, η_p_^2^ = 0.396) and the CON group (*p* = 0.001, *p* = 0.228). Simple effects for groups showed the aBMD of the CHE group was significantly higher than that of the CON group (*p* = 0.006, η_p_^2^ = 0.158) after the cheerleading practice (Figure 2B).

### Autofluorescence

For AF values, a significant time × group interaction was found (*p* = 0.001, η_p_^2^ = 0.209) (Figure 2C). Simple effects analysis for time showed a significant decrease of 2.7% in the CHE group (*p* = 0.004, η_p_^2^ = 0.175), while there was no significant change in the CON group (*p* = 0.096, η_p_^2^ = 0.062). Simple effects analysis for groups showed AF values was significantly higher in the CON group than in the CHE group (*p* = 0.048, η_p_^2^ = 0.086) after the cheerleading practice (Figure 2D).

### Physical fitness

For vertical jump, there was no significant time × group interaction effect (*p* = 0.084, η_p_^2^ = 0.066) (Figure 2E). Simple effects analysis for time showed a significant increase of 12.7% in the CHE group (*p* = 0.003, η_p_^2^ = 0.185), while there was no significant change in the CON group (*p* = 0.411, η_p_^2^ = 0.015). Simple effects analysis for groups showed vertical jump was significantly higher in the CHE group than in the CON group (*p* = 0.006, η_p_^2^ = 0.157) after the cheerleading practice (Figure 2F).

For sit-ups, there was no significant time × group interaction effect (*p* = 0.203, η_p_^2^ = 0.037) (Figure 2G). Simple effects analysis for time showed no significant increase in both the CHE group and the CON group. Simple effects analysis for groups showed sit-ups was significantly higher in the CHE group than in the CON group (*p* = 0.006, η_p_^2^ = 0.158) (Figure 2H).

### Correlation analysis

**FIGURE 3:**
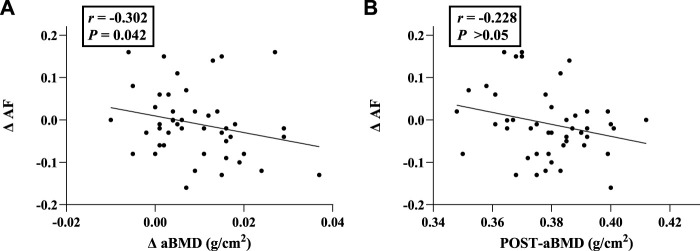
Correlation analysis between ΔAF and aBMD-related indicators. **(A)** Correlation of ΔAF with *Δ* aBMD; **(B)** Correlation of ΔAF with aBMD (Post-exercise). Abbreviations: aBMD, areal bone mineral density; AF, autofluorescence; ΔAF, change of AF value; ΔaBMD, change of aBMD.

As shown in Table 4, ΔAF values were significantly negatively correlated with *Δ* aBMD (*r* = −0.302, *p* = 0.042) (Figure 3).

**TABLE 4:** Correlation analysis between ΔAF and BMD-related indicators.

	ΔAF	*P*
ΔaBMD	−0.302	0.042
aBMD (Post-exercise)	−0.228	0.128

Abbreviations: aBMD, areal bone mineral density; AF, autofluorescence; ΔAF, change of AF, value; ΔaBMD, change of aBMD.

### Mediation analysis

**FIGURE 4:**
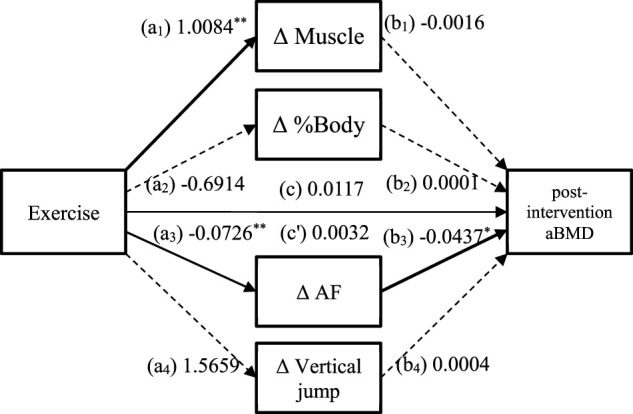
Mediated model of the relationship between exercise and post-intervention aBMD. a_1_, a_2_, a_3_, a_4_, b_1_, b_2_, b_3_, b_4_: path coefficients from the bootstrap procedure; c: the total effect of exercise on the post-intervention aBMD; c': the indirect effect of exercise on the post-intervention aBMD. The solid paths are significant (*p* < 0.05) and the dashed paths are not significant (*p* > 0.05). The covariates (physical activity, eating behaviour) are not shown on the graph for overall clarity. Abbreviations: aBMD, areal bone mineral density; AF, autofluorescence; ΔAF, change of AF value (Figure 4).

Table 5 summarises the indirect effects of exercise on post-intervention aBMD through changes in body composition, AF values, and physical fitness. Of the four mediators, the indirect effect of ΔAF values (a3b3 = 0.0032, 95% CI 0.0002–0.0079) was significant only because the CI did not contain 0. It had a total effect of 0.0117 and a direct effect of 0.0085, with the indirect effect accounting for 27.4% of the total effect. This suggests that 27.4% of the effect of exercise on post-intervention aBMD was mediated through ΔAF values (Figure 4).

**TABLE 5:** Mediated analysis of the relationship between exercise and post-intervention aBMD.

IV	Mediators	DV	IDEs	95%	CI
Exercise	Δ Muscle mass	post-intervention aBMD	−0.0016	−0.0067	0.0029
	Δ %body fat		0.0000	−0.0012	0.0016
	Δ AF		0.0032	0.0002	0.0078
	Δ Vertical jump		0.0006	−0.0013	0.0029

Physical activity and eating behavior at baseline were added as covariates in all models. Abbreviations: IV, independent variables; DV, dependent variable; IDE, indirect effect; aBMD, areal bone mineral density; AF, autofluorescence; ΔAF, change of AF, value.

## Discussion

Our primary finding was a significant increase in aBMD and a significant decrease in AGEs after 14 weeks of cheerleading practice. We observed an increase in vertical jumps and sit-ups in the cheerleading group. Moreover, the change in aBMD was found to be negatively correlated with the change in AF values, and changes in AF values mediated part of the effect of exercise on aBMD. Our results suggest that exercise could improve bone health in female adolescents and that this could be due to the reduction in AGEs levels in the body.

In this study, we found significant improvements in aBMD following cheerleading practice, which is similar to the results of other studies on using exercise to improve bone health (Zhao et al., 2014; Al et al., 2019; Perkins et al., 2019). Cheerleading evolved from competitive gymnastics and includes many jumping movements, and Zhao et al. (Zhao et al., 2014) found that using jumping movements as the main form of exercise significantly increased BMD in specific areas, such as the femoral neck and greater trochanter. In addition to this, cheerleading is also a typical aerobic exercise. Al et al. (2019) conducted aerobic exercise training with 65 female participants for a total of 60 min, three times a week for 12 weeks, and found that the aerobic exercise practice significantly increased the participants’ BMD levels. Exercise probably increases bone mineral density in several ways: 1. Cheerleading has a physiological impact benefit on human bone in the upper and lower extremities, where bone tissue is stretched to activate the Wnt/β-catenin signaling pathway through cell-localized mechanosensors, leading to bone formation. 2. Irisin increases. Irisin is a hormone secreted by the skeletal muscles during exercise. Studies have shown that in healthy populations, irisin levels are positively associated with bone mineral status and circulating osteocalcin (Colaianni et al., 2019). Bone mineral status and circulating osteocalcin play an important role in bone formation, suggesting that irisin may increase BMD by increasing bone mineral status and osteocalcin. In addition, irisin can induce MC3T3-E1 osteoblast growth at the junction of the skeletal muscle and the bone (Zhang et al., 2017). Therefore, cheerleading would provide a non-medical means to improve peak bone mass in adolescents.

This study observed a significant decrease in the AF values of the cheerleading group after 14 weeks of intervention with cheerleading. The AF value is an indicator used to evaluate the level of accumulation of AGEs in the body. At baseline, although the difference was not significant, the cheerleading group also had a slightly higher mean level of AGEs than the control group, while after the exercise, the cheerleading group had a significantly lower level of AGEs than the control group. This suggests that cheerleading exercises were effective in reversing the accumulation of AGEs levels in the body. The same results were found in a similarly designed study by Karine et al. (Rodrigues et al., 2018), who found that after 3 months of aerobic exercise practice, the higher levels of AGEs in previously physically inactive human immunodeficiency virus-infected individuals decreased to the same level as those in normal individuals. All this evidence suggests that lack of physical activity may lead to higher levels of AGEs accumulation, and that exercise may be effective in reducing AGEs accumulation, returning them to normal levels, and preventing the risk of chronic diseases.

There are three main ways in which AGEs are derived in humans: 1) exogenous intake into the body (e.g., the ingestion of foods containing high levels of AGEs); 2) endogenous formation and retention in the body (e.g., the carbonyl group of reducing sugars or aldehydes combined with lysine and arginine amino acid residues); 3) unhealthy lifestyles such as sedentary behavior, lack of exercise (Strizich et al., 2018), smoking, and long-term alcohol intake can lead to the production of AGEs (Hayashi et al., 2013; Leung et al., 2016; Lopez-Moreno et al., 2017). Researchers have suggested that younger people are vulnerable to the effects of AGEs, with Putte et al. (Van Putte et al., 2016) finding that AGEs begin to accumulate in people as young as 20 years old, and then seemingly increase steadily. Exercise probably reduces the accumulation of AGEs in the body in several ways. 1. Exercise helps alleviate hyperglycemia. Exercise has been found to alleviate hyperglycemia associated with obesity by improving insulin resistance (Reddy et al., 2019), and hyperglycemia itself might be a direct source of AGEs production and accumulation. 2. Exercise enhances the ability of the kidneys to clear AGEs. The kidneys’ proximal tubular cells play an important role in the disposal of plasma AGEs (Shen et al., 2020), and aerobic and resistance exercises significantly enhance the kidneys’ filtration ability, and improve kidney function (Wu et al., 2020). Thus, the kidneys could clear AGEs more efficiently. 3. Anti-inflammatory effects of exercise. The body’s inflammatory response will activate relevant immune cells, including macrophages and dendritic cells, and induce a switch in the metabolism of these inflammatory cells towards glycolysis. During glycolysis, the precursor substances of AGEs, methyglyoxal, and glyoxal, are produced. Exercise has an anti-inflammatory effect (Sohail et al., 2019), and thus helps to reduce the accumulation of AGEs associated with inflammation.

This study found that changes in aBMD were negatively correlated with changes in AF values and that changes in AF values mediated part of the effect of exercise on of aBMD. This suggests that the elevation in aBMD resulting from exercise may be associated with a decrease in AGEs. Previous studies have suggested that exercise could improve BMD by increasing beneficial mechanical stress on the skeleton and promoting irisin, which improves bone health (Saito and Marumo, 2010). Our study provides evidence on the possible mechanisms by which exercise improves bone health by reducing AGEs levels. AGEs have harmful effects on bone health. Studies have shown that AGEs reduce bone strength and density in humans due to increased non-enzymatic cross-linking (Yamamoto and Sugimoto, 2016) and the induction of apoptosis in osteoblasts via the ages/rage/caspase-3 signaling pathway (Liu et al., 2016). However, exercise can significantly reduce the high levels of AGEs accumulated in the body, and bring them back to the normal range, while at the same time, an increase in aBMD has been observed in this period. Therefore, the evidence from this study suggests that exercise could increase bone mineral density by reducing the accumulation of AGEs in the body, and that a reduction in AGEs is one of the mechanisms by which exercise improves bone health.

This study showed that after 14 weeks of cheerleading practice, the cheerleading group had a significantly higher vertical jump and sit-ups than the control group. Previous studies have found that exercises such as cheerleading, which involves a lot of running and jumping, can significantly improve lower limb and core muscle strength (Wisloff et al., 2004). The reasons for this might be the following. Firstly, cheerleading involves many movements such as kicks, squats, and big jumps, and is beneficial for the growth of muscles (Wisloff et al., 2004). Secondly, the large number of movement changes during the limited duration of cheerleading also helps to increase the neural control of muscles (Soendenbroe et al., 2022). Thirdly, the complex movements involved in cheerleading also improve the ability of the participants to synergistically contract the various muscle groups.

The strength of this study is that, to our knowledge, it is the first to examine both the effects of exercise on bone health and the levels of AGEs. Secondly, we have used an intelligent all-in-one machine to conduct the physical fitness test, with intelligent human voice instructions and prompts, thus avoiding the bias of manual measurements. At the same time, there were several limitations to this study. Firstly, the number of outdoor exercise practices was less per week. However, the longer durations of each practice and the online practices compensate for this to some extent. Secondly, this study only measured aBMD in participants’ carpal bones, and future studies are encouraged to test participants for whole-body aBMD. Thirdly, although the participants were theoretically informed about dietary intake during the outdoor practice, we did not systematically control and track participants’ dietary intakes during the study. Therefore, it is suggested that future studies conduct a comprehensive assessment of participants’ diets.

## Conclusion

In conclusion, cheerleading practice increased aBMD and physical fitness in female adolescents, and also decreased the levels of AGEs. Further analysis revealed that changes in aBMD were negatively correlated with changes in AGEs, and the effect of exercise on aBMD is partly mediated by AGEs. These findings suggest that cheerleading may be an effective non-pharmacological intervention to increase aBMD by reducing the accumulation of AGEs in female adolescents. Future studies should investigate the effects of exercise on aBMD and AGEs when combined with more stringent dietary control.

## Data Availability

Data are available by contacting the corresponding author upon reasonable request.
